# Nosocomial Outbreaks Caused by *Leuconostoc mesenteroides* subsp. *mesenteroides*

**DOI:** 10.3201/eid1406.070581

**Published:** 2008-06

**Authors:** Germán Bou, Jesús Luis Saleta, Juan Antonio Sáez Nieto, Mar Tomás, Silvia Valdezate, Dolores Sousa, Francisco Lueiro, Rosa Villanueva, Maria Jose Pereira, Pedro Llinares

**Affiliations:** * Complejo Hospitalario Universitario Juan Canalejo, La Coruña, Spain; † Centro Nacional de Microbiología, Majadahonda, Madrid, Spain

**Keywords:** Leuconostoc, outbreak, nosocomial setting, parenteral nutrition, risk factors, vancomycin and antibiotic resistance, dispatch

## Abstract

From July 2003 through October 2004, 42 patients became infected by strains of *Leuconostoc mesenteroides* subsp. *mesenteroides* (genotype 1) in different departments of Juan Canalejo Hospital in northwest Spain. During 2006, 6 inpatients, also in different departments of the hospital, became infected (genotypes 2–4). Parenteral nutrition was the likely source.

*Leuconostoc* species are catalase-negative, gram-positive microorganisms with coccoid morphology (*1*). In 1985, Buu-Hoi et al. (*2*) reported the first cases of *Leuconostoc* infection in humans. Since then, *Leuconostoc* spp. have been implicated in a variety of infections (*3*–*8*), particularly in patients being treated with vancomycin and in immunocompromised patients. However, these species have never previously been considered as agents that cause severe hospital outbreaks that threaten the lives of large numbers of persons.

Between July 2003 and October 2004, and between August and November 2006, 42 and 6 patients, respectively (Figure 1), in the Juan Canalejo Hospital (a tertiary-level, 1,400-bed hospital serving a population of 516,000 in La Coruña, northwest Spain) became infected by a strain of *Leuconostoc mesenteroides* subsp. *mesenteroides* (LM). The patients had been admitted to 13 different, physically separated departments in the hospital (3 different hospital buildings), and 11 of the 48 were newborns. The aims of the present study were to characterize the epidemiologic features of the outbreak and to determine the risk factors associated with the infection.

**Figure 1:**
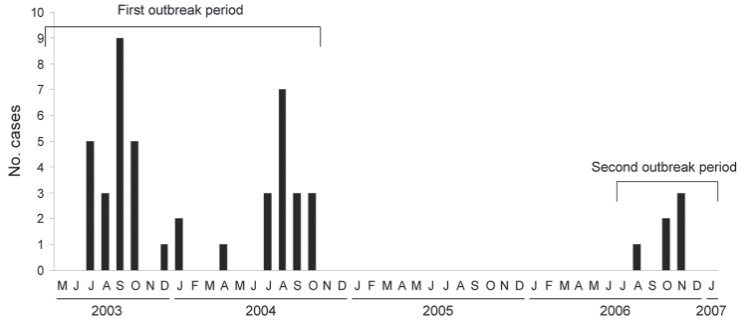
Epidemic curve of distribution of *Leuconostoc*-infected patients throughout the period of study. Two different outbreak periods were detected, July 2003 through October 2004 (42 patients) and August through November 2006 (6 patients). The first outbreak period was caused by a single epidemic strain and the second one was caused by 3 different strains.

## The Study

All bacterial isolates related to the outbreaks (1 per patient) were obtained from clinical samples. The strains were identified phenotypically by rapid ID 32 STREP (bioMérieux, Marcy l’Etoile, France), which yielded profile 22025001100 (*Leuconostoc* spp. 99.9%) and BIOLOG GP2 panels (Biolog, Hayward, CA, USA) (98%, T = 0.708). The results were confirmed by 16S rDNA sequence analysis, by a previously reported method (*9*), and the analysis of 1,420–1,500 bp showed 99% probability that the species were LM, when compared with GenBank database sequences.

Antimicrobial drug susceptibility was determined by microdilution, with DadeMicroscan system (Baxter Health Care, West Sacramento, CA, USA), and MICs were confirmed by E-test (AB Biodisk, Solna, Sweden). For interpretation of antimicrobial drug susceptibility, Clinical and Laboratory Standards Insitute criteria (*10*) for *Leuconostoc* spp. or when appropriate *Streptococcus* spp. other than *S. pneumoniae*, were used. The antimicrobial drug susceptibility profiles were almost identical for all genotypes and showed susceptibility to penicillin and gentamicin (MICs of 0.25 mg/L and <2 mg/L, respectively) and to levofloxacin, tetracycline, quinupristin-dalfopristin, linezolid, daptomycin, erythromycin, clindamycin, and chloramphenicol.

**Figure 2:**
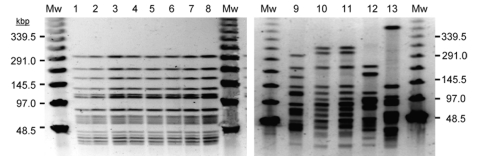
Band pattern obtained by pulsed-field gel electrophoresis of selected *Leuconostoc mesenteroides* subsp. *mesenteroides* (LM) isolates. Mw, molecular weight marker at indicated sizes; lines 1 to 9, representative LM isolates from the first outbreak (genotype 1); lines 10, 11, LM isolates obtained from parenteral nutrition catheter and blood from the same patient (genotype 2) and identical to those from 3 different patients infected in the second outbreak (data not shown); lines 12, 13, LM isolates from 2 different patients involved in the second outbreak (genotypes 3 and 4)

A pulsed-field gel electrophoresis (PFGE) technique was used to assess the possibility of a clonal relationship among the 48 LM strains. Genomic DNA was extracted, restricted with ApaI, and electrophoresed with CHEF-DRIII apparatus (Bio-Rad Laboratories, Richmond, CA, USA). The isolates were classified epidemiologically, according to published criteria (*11*). No differences in the band profile were observed among the 42 strains of the first outbreak (genotype 1). Analysis of the 6 strains isolated in the 2006 outbreak showed different DNA band patterns from those corresponding to genotype 1 (Figure 2). Of the 6 isolates, 4 shared the same genotype, designated genotype 2, whereas the remaining 2 isolates showed 2 new genotypes (genotypes 3 and 4). One LM strain, isolated from the parenteral nutrition catheter of a patient involved in the 2006 outbreak (genotype 2), was identical to those isolated from blood of the same patient (Figure 2) and from 3 other patients involved in the 2006 outbreak (data not shown).

Most of the 42 patients infected with LM genotype 1 in the first outbreak displayed severe underlying diseases (Table 1); 9 of the patients died, and 3 of the deaths (7.1%) were directly related to the *Leuconostoc* infection. The bacterial isolates were isolated from blood (52.1%), catheter (21.8%), or both (26.1%).

**Table 1:** Clinical features of the case-control patients in the first outbreak of *Leuconostoc mesenteroides* subsp. *mesenteroides* infection, La Coruña, Spain, 2003–2004

Diagnosis*	No. cases (%), n = 42	No. controls (%), n = 61
Newborns	11 (26.2)	17 (27.9)
Adults (>1 y)	31 (73.8)	44 (72.1)
Tumors		
Solid	9 (21.4)	13 (21.31)
Leukemia/lymphoma/myeloma	1/5/0 (2.38/11.9/0)	3/4/3 (4.92/6.56/4.92)
Digestive tract disease		
Pancreatitis	3 (7.14)	0
Necrotizing enterocolitis	2 (4.76)	1 (1.64)
Ulcerous colitis/Crohn disease	1/1 (2.38/2.38)	0/1 (0/1.64)
Cholecystitis	2 (4.76)	1 (1.64)
Bowel perforation	3 (7.14)	0
Bowel atresia	2 (4.76)	1 (1.64)
Bowel fistula	2 (4.76)	1 (1.64)
Prematurity	3 (7.14)	11 (18.03)
Infections†	3 (7.14)	2 (3.28)
Cardiopathy	2 (4.76)	3 (4.92)
Chylothorax	4 (9.52)	0
Brain vascular disease	3 (7.14)	8 (13.11)
Immunosupression	18 (48.9)	36 (59.0)
Others	3 (7.14)	9 (14.75)

*Each patient may have >1 diagnosis. †Concomitant with *Leuconostoc* infection.

To assess risk factors related to acquisition of LM strains, we performed a case–control study. The first 42 patients (2003–2004) were designated as case-patients. Control-patients (n = 61) were randomly selected among remaining patients with another nosocomial infection caused by a non–*Leuconostoc* spp. microorganism isolated from a catheter, blood, or both, who were admitted to the same department and at the same time as the patients defined as case-patients. The variables analyzed are shown in Table 2.

**Table 2:** Model for predicting infection by *L. mesenteroides* subsp. *mesenteroides* (LM)*,* 2003–2004, La Coruña, Spain*

Variable	Cases, n = 42			Controls, n = 61		Crude OR† (95% CI)	Adjusted OR (95% CI)	p value
	No. (%)	Mean (SD)		No. (%)	Mean (SD)			
Age, y		34.3 (28.2)			44.4 (31.7)	0.99 (0.98–1.0)		
Time between admission and infection		33.5 (38.4)			37.5 (100.9)	0.999 (0.994–1.0)	NS	
Charlson score		2.94 (2.13)			4.28 (2.38)	0.76 (0.61–0.96)	NS	
Previous surgery	29 (69)			23 (37.7)		3.7 (1.6–8.5)	NS	
Previous infections	31 (73.8)			15 (24.6)		8.6 (3.5–21.3)	4.2 (1.2–14.7)	0.023
Previous antimicrobial drug therapy	37 (88.1)			42 (68.9)		3.3 (1.1–9.9)	NS	
Teicoplanin	12 (28.6)			4 (6.6)		5.7 (1.7–19.2)	NS	
Vancomycin	5 (11.9)			3 (4.9)		2.6 (0.6–11.6)		
Central venous lines	39 (92.9)			41 (67.2)		6.3 (1.7–23.0)	NS	
Sex								
Male	24 (57.1)			41 (67.2)		0.7 (0.3–15)		
Urinary catheter	28 (66.7)			21 (34.4)		3.7 (1.6–8.6)	NS	
Enteral nutrition	18 (42.9)			23 (37.7)		1.2 (0.6–2.8)		
Parenteral nutrition	40 (95.2)			26 (42.6)		26.9 (6–121.6)	27.8 (5.5–141.1)	<0.000
Blood transfusion	24 (57.1)			31 (50.8)		1.3 (0.6–2.8)		
Intubation	18 (42.9)			17 (27.9)		1.9 (0.8–4.4)		
Tracheostomy	4 (9.5)			3 (4.9)		2.0 (0.4–9.6)		
Treatment with steroids	8 (19)			12 (19.7)		1 (0.4–2.6)		
Alteration of gastrointestinal barrier‡	29 (69)			30 (49.2)		2.3 (1.0–5.3)	NS	

*Values for 42 case patients and 61 control patients. SD, standard deviation; OR, odds ratio; CI, confidence interval; NS, variable did not meet criterion for remaining in the multivariate model. All these variables were considered as potential risk factors for LM infection. The LM infection was considered as an outcome variable. †Predictor variables with p<0.10 in univariate analysis were included in the multivariate model to enable simultaneous adjustment. ‡Any process that modifies the gastrointestinal barrier (inflammation, atresia, resection, obstruction).

Nosocomial infection criteria were those previously established by the Centers for Disease Control and Prevention (Atlanta, GA, USA) (*12*). A multiple logistic regression model was developed to identify potential independent factors associated with acquisition of LM strains. Predictor variables with p<0.10 in univariate analysis were included in the multivariate model to enable adjustment. Statistical analyses were conducted with SPSS 14.0 software (SPSS Inc., Chicago, IL, USA).

According to the multivariate analysis, previous infections (38.2% were bacteremias) (odds ratio [OR] = 4.2) and parenteral nutrition (OR = 27.8) were associated with *Leuconostoc* spp. infection (Table 2). After the case–control study, parenteral nutrition was suspected to be the source of the outbreak.

All case-patients received parenteral nutrition, with the exception of 2, although they received enteral nutrition. Parenteral nutrition is a putative source of the infection because all parenteral and enteral nutrition bags are prepared in the central hospital pharmacy and then distributed to the different medical units in the hospital. This possibility was further supported by 1 finding: PFGE analysis of isolates obtained from a parenteral nutrition catheter connected to a patient during the second outbreak yielded the same genotype as the isolates obtained from blood from the same patient (Figure 2) and from another 3 physically separated, infected patients. The physical distance between these patients as well as the impossibility of retrograde displacement of the bacterial isolate from patient’s blood makes it unlikely that the LM strain was acquired by contamination from the blood and indicates parenteral nutrition as the main source of LM transmission in the hospital outbreak. Microbiologic controls of parenteral nutrition were reinforced during the second outbreak, and as stated, only 6 cases were detected. Moreover, during the second outbreak, microbiologic analysis of environmental samples as well as samples from the digestive tract, skin, and throat of all patients involved did not yield any *Leuconostoc* strains.

Parenteral nutrition controls performed in the hospital pharmacy department are now routinely assayed for LM isolation. Since the last LM outbreak in November 2006, no cases of *Leuconostoc*-associated bacteremia have been reported in the hospital.

## Conclusions

That 42 LM isolates from the first outbreak shared the same genotype and 4 of 6 isolates in the second outbreak also shared the same (another) genotype rules out the possibility of endogenous infections among patients and suggests a common source for each outbreak. The occurrence of cases in patients in areas that were physically separated rules out the possibility of indirect patient-to-patient spread through the hands of healthcare workers or contaminated hospital equipment (different departments do not share healthcare workers and equipment).

Enteral and parenteral nutrition has previously been described (*13*,*14*) as a risk factor associated with *Leuconostoc*-infections, although no microbiologic evidence was provided in any of the studies. With regard to previous infections in the multiple logistic regression model, this may be related to the alteration of the immune system caused by the microorganism that caused the previous infections. This alteration may play a role facilitating the subsequent *Leuconostoc* spp. infection.

Two previous reports have described hospital transmission of *Leuconostoc* spp (*7*,*15*); both outbreaks affected a small number of patients, and no epidemiologic studies were conducted to clarify the genetic relationship among the bacterial strains involved or the source of the nosocomial infection. Although up to 88 cases of *Leuconostoc* infection have been reported in the scientific literature in the past 25 years, these cases may not be comparable to those reported here, the largest nosocomial outbreak caused by *Leuconostoc* spp. worldwide.

This outbreak highlights the importance of LM as an emerging hospital pathogen in patients with underlying diseases and in whom parenteral nutrition may be the source of the initial infection and its spread. Every infection with LM could be a yet undetected outbreak and should result in an investigation that focuses on parenteral nutrition or products manufactured in a centralized hospital pharmacy.
